# New data on aphid fauna (Hemiptera, Aphididae) in Algeria

**DOI:** 10.3897/zookeys.319.4340

**Published:** 2013-07-30

**Authors:** Malik Laamari, Armelle Coeur d’Acier, Emmanuelle Jousselin

**Affiliations:** 1 LATPPAM Laboratory, Department of Agronomy, Institute of Agronomic and Veterinary Sciences, University of Batna, Algeria; 2 INRA, UMR CBGP (INRA/IRD/Cirad/Montpellier SupAgro), Campus International

**Keywords:** Aphids, biodiversity, Algeria, Maghreb, North Africa

## Abstract

A survey of aphids was carried out during the period 2008-2011 in different regions of Algeria by collecting and identifying aphids and their host plants. Aphids were collected from 46 host plants. Forty-six species were reported including thirty-six species which were recorded for the first time in this country and thirty species which were recorded for the first time in the Maghreb (North Africa). This study extends the number of known Algerian aphid to 156 species.

## Introduction

The aphid fauna of North Africa has been poorly studied. One hundred and fifty eight species have been recorded from Morocco (Mimeur 1932, 1934, 1935a, 1935b, 1937, 1941, 1942, Blackman and Eastop 1994, 2000, 2006, Sekkat 1987). One hundred and three species are recorded from Tunisia (Bodenheimer and Swirsky 1957, Blackman and Eastop 1994, 2000, 2006, Ben Halima-Kamel 1991, 1995, Ben Halima-Kamel and Ben Hamouda 1993, 1998, 2004, 2005, Boukhris-Bouhachem et al. 1996, Boukhris-Bouhachem et al. 2007). Ninty nine species are listed from Egypt (Theobald 1922, Habib and El Kady 1961, Darwish 2009). Aphids in Libya are reprisented by seventy three species (Trotter 1912, 1914, Damiano 1961, 1962, Blackman and Eastop 1994, Ahmeid Al Nagar 2000, Ahmeid Al-Najar and Nieto Nefrya 1998). The Algerian aphid fauna is now partly known (Mimeur and Bernard 1944, Bodenheimer and Swirsky 1957, Remaudière and Leclant 1974, Dartigues 1993, Blackman and Eastop 1994, 2000, 2006, Laamari and Akkal 2002). Laamari et al. (2010) present a list of aphids and their host plants in Algeria. In this important publication, 120 aphid species are listed and commented/discussed. The bibliography of most papers concerning the aphid fauna of the country is provided.

## Material and methods

**Figure 1.**
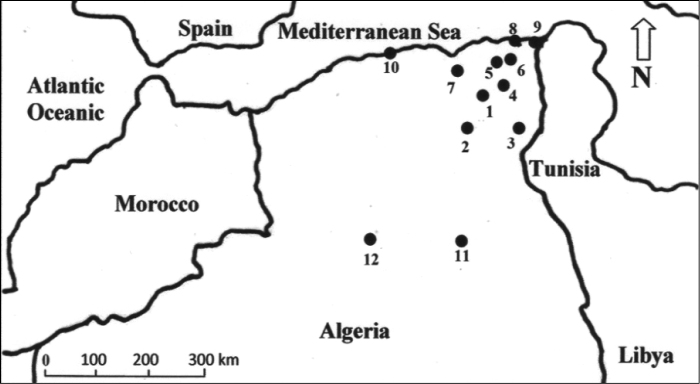
Map of the regions where samples were collected. **1** Batna **2** Biskra **3** Khenchela **4** Oum El Bouaghi **5** Constantine **6** Guelma **7** Setif **8** Annaba **9** Taref **10** Algiers **11** Ouargla **12** Ghardaia

The regions choosen for sample collection belonged to different bioclimatic stages. The regions of Annaba, Tarf and Algiers are located on the Mediterranean coast and are characterized by a humid and sub humid climate. Other regions (Guelma, Constantine, Setif and Oum El Bouaghi) are located on the high plateaus and high plains, where cereal crops are cultivated (semi arid climate). Khenchela, Batna and Biskra are located on the slopes north and south of the Saharan Atlas. Their natural vegetation is dominated by steppe plants. Ouargla and Ghardaia are located almost in the center of the Algerian Sahara (arid climate). Their natural vegetation is composed of desertic plants (Fig. 1).

This study, conducted between 2008 and 2011, considers only the new aphid species of Algeria and the species already mentioned but without specified host plants. Specimens were collected from wild and cultivated plants, tree and shrubs bearing aphid colonies. The aphids were preserved in 75% ethyl alcohol until their preparation for slide-mounting. They were identified using the keys of Blackman and Eastop (1994, 2000), Nieto Nafria et al. (2002, 2005) and Remaudière et al. (1985). The nomenclature used was that proposed by Remaudière and Remaudière (1997). The aphid preserving techniques are mainly based on the method of Hille Ris Lambers (1950). The majority of the studied and identified specimens were deposited in the insect collection of the Department of Agronomy, University of Batna (Algeria) and a minor part of aphids were deposited in the collection of the National Institute of Agronomic Research (INRA) at the CBGP in Montpellier, France.

## Results

During this study, 320 samples were collected from the investigated regions. A total of 46 aphid species were reported including 36 species which were recorded for the first time in the country and 30 species which were recorded for the first time in the Maghreb (North Africa). The presence of 10 species already reported from Algeria was confirmed. Aphid species were listed in systematic category alphabetically, including the host plant and region for each aphid species (Table 1).

**Table 1.** List of aphid species present/found in Algeria.

**Aphid species**	**Host plants**	**Regions**
**Acyrthosiphon kondoi Shinji, 1938	*Fagonia glutinosa* Delile	Biskra
*Aphis acanthoidis* (Börner, 1940)	*Carduncellus plumosus* Pomel	Khenchela
**Aphis acetosae Linnaeus, 1761	*Rumex crispus* L.	Batna
**Aphis astragali Ossiannilsson, 1959	*Astragalus armatus* Willd	Batna
**Aphis balloticola Szelegiewicz, 1968	*Balota nigra* L.	Batna
**Aphis cytisorum Hartig, 1841	*Calicotome villosa* (Poiret) Link	Guelma
*Aphis illinoisensis Shimer, 1866	*Vitis vinifera* L.	Taref, Batna
**Aphis impatientis Thomas, 1878	*Rosa damascena* Mill.	Biskra
**Aphis intybi Koch, 1855	*Cichorium intybus* L.	Taref
**Aphis medicaginis Koch, 1854	*Ononis angustissima* Lam.	Khenchela
**Aphis middletonii Thomas, 1879	*Taraxacum officinale* F.H. Wigg	Khenchela
**Aphis potentillae Nevsky, 1929	*Potentilla reptans* L.	Batna
**Aphis salviae Walker, 1952	*Lavandula multifida* L.	Batna
**Aphis stroyani Szelegiewicz, 1961	*Picris echioides* L.	Guelma
**Aphis thomasi (Börner, 1950)	*Knautia arvensis* (L.) J.M. Coult	Batna
**Aphis umbrella (Börner, 1950)	*Malva sylvestris* L.	Batna
*Aphis verbasci* Schrank, 1801	*Verbascum thapsus* L.	Batna
*Brachycaudus persicae (Passerini, 1860)	*Ononis natrix* L.	Batna
*Brachyunguis tamaricis (Lichtenstein, 1885)	*Tamarix gallica* L.	Biskra
*Chaitophorus leucomelas Koch, 1854	*Populus alba* L.	Guelma
*Cinara cedri* Mimeur, 1936	*Cedrus atlantica* (Endl.) G. Manetti ex Carrière	Batna
**Cinara juniperi (de Geer, 1773)	*Juniperus oxycedrus* L.	Batna
*Clypeoaphis suaedae (Mimeur, 1934)	*Suaeda fruticosa* Forsk.	Biskra
*Capitophorus elaeagni* (del Guercio, 1894)	*Silybum marianum* Garten, *Lawsonia inermis* L.	Biskra
*Dysaphis tulipae* (Boyer de Fonscolombe, 1814)	*Iris germanica* L.	Batna
*Greenidea ficicola Takahashi, 1921	*Ficus retusa* L.	Algiers
**Indiochaitophorus furcatus Verma, (1970)	*Ulmus campestris* L.	Biskra
**Liosomaphis berberidis (Kaltenbach, 1843)	*Achillea santolina* L.	Batna
**Macrosiphoniella grandicauda Tak. & Mor.,1963	*Artemisia herba-alba* Asso	Biskra
*Nasonovia ribisnigri* (Mosley, 1841)	*Andryala integrifolia* L., *Geranium pusillum* L.	Batna
**Pterocomma pilosum Buckton, 1879	*Salix pedicellata* Desf.	Batna
**Semiaphis heraclei (Takahashi, 1921)	*Torilis nodosa* (L.) Gaertn.	Khenchela
*Sipha maydis* Passerini, 1860	*Digitaria sanguinalis* (L.) Scop	Guelma
**Sitobion lambersi David, 1956	*Bromus squarrosus* L.	Batna
**Stomaphis pini Takahashi, 1920	*Pinus halepensis* Mill.	Batna
**Therioaphis riehmi (Börner, 1949)	*Trigonella anguina* Delile	Biskra
**Tinocallis takachihoensis Higuchi, 1972	*Ulmus campestris* L.	Biskra
**Uroleucon ambrosiae (Thomas, 1878)	*Carthamus lanatus* L.	Batna
**Uroleucon aeneum (Hille Ris Lambers, 1939)	*Onopordum Illyricum* L.	Batna
**Uroleucon bifrontis (Passerini, 1879)	*Dittrichia viscosa* (L.) Greuter	Khenchela
**Uroleucon carthami (Hille Ris Lambers, 1948)	*Carthamus lanatus* L.	Batna
**Uroleucon chrysanthemi (Oestlund, 1886)	*Calendula arvensis* L.	Khenchela
*Uroleucon compositae* (Theobald, 1915)	*Borago officinalis* L.	Batna
*Uroleucon erigeronense* (Thomas, 1878)	*Erigeron canadensis* L., *Artemisia herba-alba* Asso	Khenchela
**Uroleucon inulicola (Hille Ris Lambers, 1939)	*Senecio vulgaris* L.	Batna
*Uroleucon pilosellae* (Börner, 1933)	*Leontodon hispidus* L.	Biskra

* = species reported for the first time in Algeria

** = species reported for the first time in the Maghreb

## Discussion

With 46 species, this survey constitutes the most important contribution to the knowledge on aphid diversity in Algeria. Organization of the similar local studies would to play an important role in the applied entomological studies and may add more species to Algerian aphid fauna. There is a very large volume of literature about all the major pest aphid species and two factors that have the greatest influence on intraspecific variation in aphids: the life cycle and the host plant. Among the species inventoried, *Aphis illinoisensis* is the aphid that has the greatest agricultural importance. This invasive aphid was reported for the first time in the Mediterranean from southern Turkey in 2002, and identified as a new possible threat to the respective grape-growing areas (Remaudière et al. 2003). A general historical set of invasive grape aphid detection is as follows: 2002 in southern Turkey (Remaudière et al. 2003), 2005 in Crete - Greece (Tsitsipis et al. 2005), 2007 in Israel (Barjadze and Ben-Dov 2011), 2009 in Tunisia (Ben Halima-Kamel and Mdellel 2010). In Algeria this aphid was detected for the first time in 2007 in several regions of viticulture (Laamari and Coeur d´Acier 2010).

*Greenidea ficicola* is considered as another invasive species. It was encountered for the first time in 2007 on *Ficus nitida* in Tunisia (Ben Halima-Kamel 2009). In Algeria was collected in April, 2008.

A total of 34 aphid species were collected on the steppe plants specific of the Saharian Atlas. This mountain range forms the boundary between the northern (Mediterranean area) and southern (African area) of Algeria. It is home to many endemic plants, which may harbour very specific and uncommon aphid species. All aphid species reported as new to Algeria and North Africa were found in this transition area (Batna, Biskra and Khenchela regions).

## Conclusion

In this study, 36 aphid species were reported for the first time in Algeria, increasing the number of species known to be present in this country to 156. Given the high level of climatic and plant diversity in Algeria, the expansion of prospect activities to a larger number of plant species and environments would undoubtedly provide a more accurate picture of the Algerian aphid fauna and would increase the number of species known to be present in this country. Furthermore, prospect studies in the Sahara and steppe zones, which are know to have a highly endemic flora, might lead to the description of species new to science.
